# Using Genetic Distance from Archived Samples for the Prediction of Antibiotic Resistance in *Escherichia coli*

**DOI:** 10.1128/AAC.02417-19

**Published:** 2020-04-21

**Authors:** Derek R. MacFadden, Bryan Coburn, Karel Břinda, Antoine Corbeil, Nick Daneman, David Fisman, Robyn S. Lee, Marc Lipsitch, Allison McGeer, Roberto G. Melano, Samira Mubareka, William P. Hanage

**Affiliations:** a Division of Infectious Diseases, University of Toronto, Toronto, Canada; b Harvard T. H. Chan School of Public Health, Boston, Massachusetts, USA; c Ottawa Hospital Research Institute, Ottawa, Canada; d University Health Network, Toronto, Canada; e Harvard Medical School, Boston, Massachusetts, USA; f Dalla Lana School of Public Health, University of Toronto, Toronto, Canada; g Public Health Ontario Laboratory, Toronto, Canada; h Department of Laboratory Medicine and Pathobiology, University of Toronto, Toronto, Canada

**Keywords:** empirical antibiotics, antibiotics, genomics, Gram-negative bacteria, antibiotic-resistant organisms, rapid diagnostics

## Abstract

The rising rates of antibiotic resistance increasingly compromise empirical treatment. Knowing the antibiotic susceptibility of a pathogen’s close genetic relative(s) may improve empirical antibiotic selection. Using genomic and phenotypic data for Escherichia coli isolates from three separate clinically derived databases, we evaluated multiple genomic methods and statistical models for predicting antibiotic susceptibility, focusing on potentially rapidly available information, such as lineage or genetic distance from archived isolates. We applied these methods to derive and validate the prediction of antibiotic susceptibility to common antibiotics. We evaluated 968 separate episodes of suspected and confirmed infection with Escherichia coli from three geographically and temporally separated databases in Ontario, Canada, from 2010 to 2018. Across all approaches, model performance (area under the curve [AUC]) ranges for predicting antibiotic susceptibility were the greatest for ciprofloxacin (AUC, 0.76 to 0.97) and the lowest for trimethoprim-sulfamethoxazole (AUC, 0.51 to 0.80). When a model predicted that an isolate was susceptible, the resulting (posttest) probabilities of susceptibility were sufficient to warrant empirical therapy for most antibiotics (mean, 92%). An approach combining multiple models could permit the use of narrower-spectrum oral agents in 2 out of every 3 patients while maintaining high treatment adequacy (∼90%). Methods based on genetic relatedness to archived samples of E. coli could be used to predict antibiotic resistance and improve antibiotic selection.

## Abstract (precis)

The rising rates of antibiotic resistance increasingly compromise empirical treatment. Knowing the antibiotic susceptibility of a pathogen’s close genetic relative(s) may improve empirical antibiotic selection. Using genomic and phenotypic data for Escherichia coli isolates from three separate clinically derived databases, we evaluated multiple genomic methods and statistical models for predicting antibiotic susceptibility, focusing on potentially rapidly available information, such as lineage or genetic distance from archived isolates.

## INTRODUCTION

Antibiotic resistance is a major global threat to public health (1). Antibiotic-resistant organisms (AROs) and mechanisms of antibiotic resistance are selected through the use of antibiotics in humans, animals, and environments (2). The prevalence of AROs in human infections has been increasing in many regions and across many different bacterial species (1, 3). As a result, empirical antibiotic therapy (therapy administered prior to knowledge of the organism’s antibiotic susceptibility phenotype) has become increasingly challenging for both community- and hospital-acquired infectious syndromes. Inadequate empirical therapy, i.e., treatment that does not include an agent to which the etiologic pathogen is susceptible, has been associated with worse patient outcomes (4–6). Moreover, the increasing rates of antibiotic resistance in common infections lead to the more frequent use of broader-spectrum antibiotic agents, with their added toxicity and enhanced selection of antibiotic resistance by targeting a wider array of pathogenic, opportunistic, and commensal bacteria.

Reducing the time from presentation and sample collection to reporting of antibiotic susceptibility has long been touted as a potential means to improve early adequate therapy and reduce the use of antibiotic agents with unnecessarily broad activity (7, 8). The development of rapid diagnostic tests that can narrow these windows of empirical antibiotic therapy is the focus of active research, but the translation of these tests to clinical practice has been slow, due to the inconsistency between individual or combined genetic loci and the expected phenotype, the need for specialized equipment, cost, challenges of commercialization, and poor integration into the clinical work flow (7). Recently, genomic approaches have been identified as rapid diagnostic tests, offering the promise of culture-independent (and -dependent) identification of (i) species, (ii) the relationship(s) to genetic neighbors/groups/clusters from databases of known isolates, and (iii) prediction of antibiotic resistance based on the relationship(s) to genetic neighbors/groups/clusters from databases of known isolates. However, the traditional approach to predicting antibiotic resistance rests on the identification of individual resistance loci to predict the phenotype. This requires a high-quality database of resistance-causative elements and is further complicated by significant cost, large physical space requirements, complicated work flow, limited expertise, and long sequencing/bioinformatic processing times even with real-time sequencing technologies (9). On the other hand, a recently introduced alternative approach called genomic neighbor typing infers antibiotic resistance and susceptibility by identifying a sample’s closest relatives in a database of genomes with known phenotypes (10). This relies on a strong correlation between the phylogenetic group and the resistance phenotype, which is observed for many bacteria (11–13).

As neighbor typing uses all genomic data available from a given set of reads, identification of a best-match isolate or lineage (i.e., a genetically related cluster or group, such as the multilocus sequence type [ST]) can occur within minutes (as opposed to hours or days for locus-based approaches, depending upon the sequencing technology used). A proof of principle has been demonstrated for Streptococcus pneumoniae and Neisseria gonorrhoeae, with determination of resistance or susceptibility being possible within 10 min of Oxford Nanopore Technologies MinION sequencing of cultured isolates and respiratory metagenomic samples (10). Limited data also suggest that the association between the antibiotic susceptibility phenotype and lineage may also hold true for *Enterobacteriaceae* (14, 15), but this approach requires further validation. It is also unknown whether prediction of the antibiotic susceptibility phenotype based on the phenotype of the nearest genetic neighbor provides advantages over the use of the average phenotype of a broader (or higher-level) lineage (e.g., ST, clonal complex, or cluster). In order to understand the potential clinical application of these techniques, we sought to validate the association between genetic relatedness (determined using nearest neighbor and lineage markers) and the antibiotic susceptibility phenotype in the most common Gram-negative pathogen in humans, Escherichia coli.

## RESULTS

### Description of the data sets.

We collected and sequenced the genomes of 968 unique E. coli isolates from separate clinical episodes of suspected infection across three data sets. The collection and sequencing details are outlined in Materials and Methods and the supplemental material. The characteristics of each data set are shown in Table 1.

**TABLE 1:** Characteristics of the data sets

Characteristic	Value or information for:		
	Data set 1	Data set 2	Data set 3
No. of isolates (*n* = 968)	411	177	380
Collection period	2010–2015	2018	2010 and 2015
Location	Toronto, Canada (city)	Toronto, Canada (city)	Southeastern Ontario, Canada
Location type	Hospital lab	Hospital lab	Hospital lab
Inpatient or outpatient	Inpatient	In- and outpatients	In- and outpatients
Anatomic site	Blood	Urine	Variable
Sampling bias	None	None	MDR
No. (%) of isolates susceptible to the following antibiotics:			
Ciprofloxacin	297 (72)	120 (68)	118 (31)
Ceftriaxone	357 (87)	155 (88)	236 (62)
Gentamicin	355 (86)	156 (88)	229 (60)
Trimethoprim-sulfamethoxazole	292 (71)	125 (71)	79 (21)
Ertapenem	410 (99)	177 (100)	338 (89)
No. (%) of isolates with the following predominant ST:			
1193	14 (3.4)	15 (8.5)	21 (5.5)
127	15 (3.6)	7 (4.0)	3 (0.8)
131	87 (21)	36 (20)	170 (45)
38	7 (1.7)	2 (1.1)	12 (3.2)
405	9 (2.2)	1 (0.6)	11 (2.9)
648	6 (1.5)	8 (4.5)	17 (4.5)
69	22 (5.4)	13 (7.3)	23 (6.1)
73	57 (14)	20 (11)	20 (5.3)
95	58 (14)	19 (11)	12 (3.2)
Other	136 (33)	56 (32)	91 (24)

### Genetic relatedness of different data sets and antibiotic resistance phenotype.

**FIG 1:**
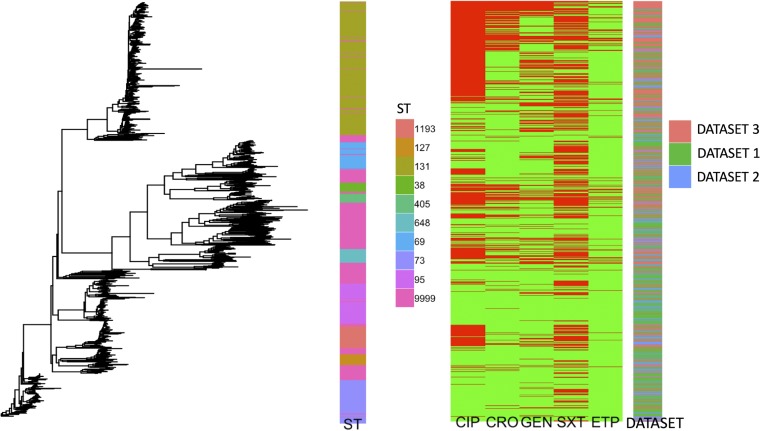
Mash tree (left), ST (middle left), phenotypic susceptibility by antibiotic (middle right), and data set (right), by individual isolate. Antibiotic susceptibility is denoted in green, and resistance is denoted in red. Abbreviations: CIP, ciprofloxacin; CRO, ceftriaxone; GEN, gentamicin; SXT, trimethoprim-sulfamethoxazole; ETP, ertapenem; ST 9999, all remaining or unknown STs.

To illustrate the linkage between relatedness and resistance, a genetic tree was constructed using Mash distances, the associated ST, the antibiotic susceptibility phenotype, and the source data set (Fig. 1). Broad genetic clusters tended to match or be nested within STs, and closely related isolates had similar antibiograms.

From the Mash tree illustrating the high-level relationships between the genomes (Fig. 1), there are clear genetic clusters that emerge. These phylogenetic groups are generally nested within specific STs, with ST131 being the most prevalent in each data set (Table 1). Unsurprisingly, there was a higher prevalence of resistance to almost all antibiotic groups in the multidrug-resistant (MDR) data set (data set 3) than in data sets 1 and 2. Despite the fact that the three data sets were separated on different scales (temporally, geographically, anatomically), they showed genetic diversity that was well distributed across different phylogroups (Fig. 1).

### ST parametric model approach (lineage based).

We calculated the areas under the curves (AUCs) and test characteristics for predicting the antibiotic susceptibility of data set 2 isolates using a parametric logistic regression model with STs as categorical predictors across a variety of derivation data sets (see Table S3 in the supplemental material). The AUCs ranged from 0.89 to 0.91 for ciprofloxacin, 0.77 to 0.80 for ceftriaxone, 0.68 to 0.75 for gentamicin, and 0.6 to 0.73 for trimethoprim-sulfamethoxazole. For ertapenem, we performed internal derivation for data set 1, data set 3, and data sets 1 and 3 combined (data set 2 could not be used, as there was 100% susceptibility to ertapenem) and found apparent and optimism-adjusted AUCs ranging from 0.7 to 0.99 and 0.67 to 0.99, respectively (Table S2).

### Cluster parametric model approach (lineage based).

We calculated the AUC and test characteristics for predicting antibiotic susceptibility (internally) for each data set with a parametric logistic regression model with clusters as categorical predictors, using a variety of derivation data sets (Table S4). AUCs ranged from 0.76 to 0.9 for ciprofloxacin, 0.69 to 0.82 for ceftriaxone, 0.66 to 0.77 for gentamicin, and 0.65 to 0.75 for trimethoprim-sulfamethoxazole. For ertapenem, we performed internal derivation for data set 1 and data set 3 and found apparent and optimism-adjusted AUCs ranging from 0.74 to 0.98 and 0.7 to 0.98, respectively (Table S2).

### ST reference database approach (lineage based).

We calculated the AUCs and test characteristics for predicting antibiotic susceptibility for isolates in data set 2 with an ST reference database, using a variety of derivation data sets (Table S5). AUCs ranged from 0.85 to 0.95 for ciprofloxacin, 0.67 to 0.85 for ceftriaxone, 0.73 to 0.83 for gentamicin, and 0.56 to 0.8 for trimethoprim-sulfamethoxazole.

### Best-genetic-match reference database approach (nearest neighbor).

We calculated the AUCs and test characteristics for predicting antibiotic susceptibility for isolates in data set 2 with a best-genetic-match reference database, using a variety of derivation data sets (Table S6). For all isolates, AUCs ranged from 0.83 to 0.92 for ciprofloxacin, 0.58 to 0.72 for ceftriaxone, 0.65 to 0.66 for gentamicin, and 0.53 to 0.63 for trimethoprim-sulfamethoxazole. For the top 25th percentile of best-match data sets, AUCs ranged from 0.85 to 0.97 for ciprofloxacin, 0.57 to 0.88 for ceftriaxone, 0.76 to 0.86 for gentamicin, and 0.54 to 0.73 for trimethoprim-sulfamethoxazole. In any method based on comparing new samples with an existing database, it is important to investigate how large the database needs to be to permit an accurate prediction. Thus, we evaluated the impact of various reference database sizes on the performance of the genetic distance approach, with the results being shown in Fig. S1.

### Summary of test characteristics across models.

**FIG 2:**
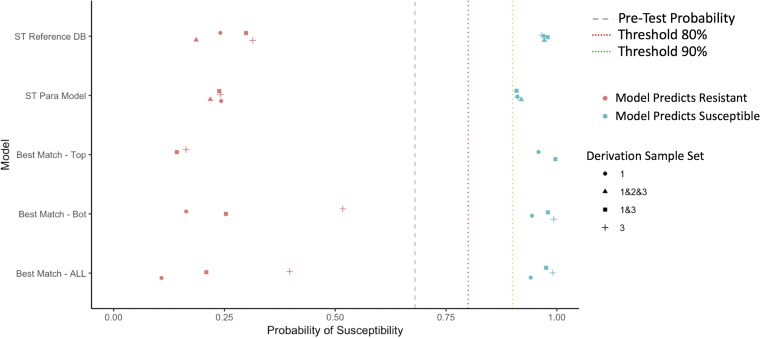
Selected posttest probabilities of ciprofloxacin susceptibility (in data set 2) based on model predictions of resistant or susceptible, by model type and derivation data set. DB, database; Para, parametric; Bot, bottom.

**FIG 3:**
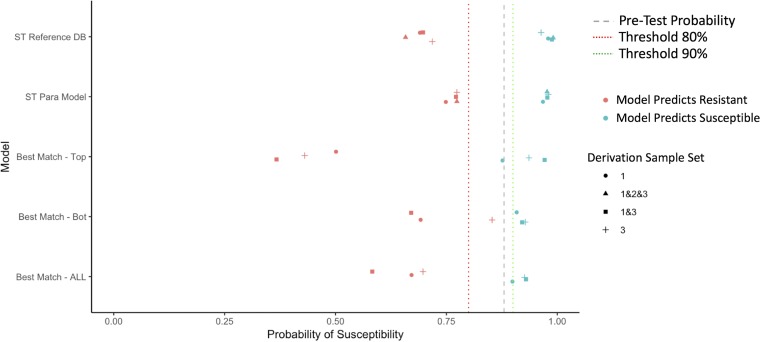
Selected posttest probabilities of ceftriaxone susceptibility (in data set 2) based on model predictions of resistant or susceptible, by model type and derivation data set.

**FIG 4:**
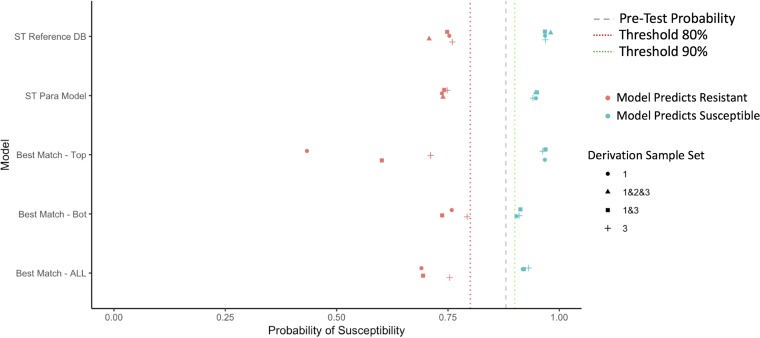
Selected posttest probabilities of gentamicin susceptibility (in data set 2) based on model predictions of resistant or susceptible, by model type and derivation data set.

**FIG 5:**
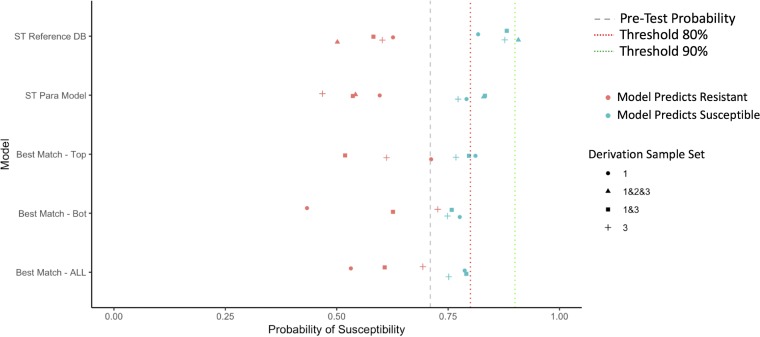
Selected posttest probabilities of trimethoprim-sulfamethoxazole susceptibility (in data set 2) based on model predictions of resistant or susceptible, by model type and derivation data set.

In Fig. 2 to 5, we summarize the posttest probabilities of susceptibility for (i) positive model predictions indicating a likely susceptible isolate (positive predictive value [PPV]) and (ii) for negative model predictions indicating a likely resistant isolate (1 − negative predictive value [NPV]). The aim of the models for which the results are presented in Fig. 2 to 5 is to shift green data points to the right (indicating that the model has correctly classified certain isolates as susceptible) and red data points to the left (indicating that the model has correctly classified certain isolates as resistant). Here we see that the models that provide the best positive and negative predictive values are the ST reference database, the best-genetic-match reference database, and combinations of the two (Table S7). We also see that for most antibiotics and models, the posttest probabilities for results indicating a susceptible result are sufficiently high (relative to syndromic thresholds) to support a recommendation of therapy. Similarly, for most antibiotics and models, the posttest probabilities of results indicating resistance is sufficiently low to support withholding therapy for a particular agent. Lastly, combined derivation data sets tend to have the most consistent model performance.

### Sequential antibiotic decision-making based upon a prespecified antibiotic cascade.

When using a sequential selection model favoring narrow-spectrum antibiotics, adequate therapy was achieved for 89 to 98% of recommendations, with 47 to 70% of the recommendations being a narrower-spectrum agent with an oral formulation (i.e., ciprofloxacin or trimethoprim-sulfamethoxazole). Spectrum scores attained with the narrow-spectrum model cascade were consistently lower than those attained with the employment of typical empirical agents (Table S7). Using an adequacy-focused cascade, adequate therapy was achieved for 98 to 100% of recommendations, with 0 to 5% of the recommendations being narrower-spectrum agents with oral formulation options. Spectrum scores were generally high but were lower than those for the most-broad-spectrum agent, ertapenem.

To summarize, when using a narrow-spectrum-focused cascade, the models yield excellent adequacy while enabling over two-thirds of recommendations to be narrow-spectrum oral agents. When using an adequacy-focused cascade, they yielded close to perfect adequacy but at the expense of using broader-spectrum agents (Table S7).

## DISCUSSION

In this study, we demonstrate that we can predict the resistance phenotype of E. coli by rapidly determining the genetic relatedness of the infecting pathogen to a database of sequenced isolates with known resistance phenotypes. We show that these relationships can be used to generate posttest probabilities of susceptibility in excess of 0.8 or 0.9, which render antibiotics to which pathogens show a high prevalence of resistance to be empirically useful (e.g., ciprofloxacin or trimethoprim-sulfamethoxazole for urinary tract infection). In essence, adoption of this approach would modify the current stages of empirical therapy to introduce a new window that is informed by genetic relatedness and information that is available in advance of standard phenotypic testing. This approach is a supplement to and not a replacement for the gold standard, phenotyping. The contribution of such a system could be to improve the quality of antimicrobial prescribing in the window between culture positivity and phenotypic testing results by (i) reducing the expected time from culture positivity to adequate treatment and (ii) reducing the duration of use of broad-spectrum agents to treat infections that could be adequately treated with a narrow-spectrum agent.

Looking at the distribution of STs across the data sets (Fig. 1; Table 1), we see that data set 3 is enriched for ST131, and this is presumably due to the intentionally biased sampling approach toward MDR. However, it means that the lineage and nearest-neighbor approaches will be able to draw sample phenotype predictions from different data sets based on genetic proximity and not simply ones most closely related on temporal, geographic, or anatomic scales.

When predicting antibiotic resistance based on lineage, the ST parametric model and cluster parametric model approaches (see Tables S3 and S4 in the supplemental material) provided reasonable discrimination for ciprofloxacin susceptibility (AUCs, 0.76 to 0.91), in keeping with existing literature (15). This emphasizes the strong association between lineage and ciprofloxacin susceptibility. In contrast, there were only modest associations for the other antibiotic classes (AUCs, 0.6 to 0.82). For all antibiotic classes, the use of combined derivation data sets (data sets 1, 2, and 3 or data sets 1 and 3) seemed to perform well most consistently, and this supports the use of an aggregated derivation data set across time, geography, and anatomic location.

The use of an ST reference database approach has the appeal of providing improved predictions for less common STs. When considering only those isolates with a matching ST in the reference database, the discrimination of this approach paralleled and sometimes exceeded that of the ST and cluster parametric models (Table S5). The notable downside to this approach is the inability to provide predictions for sequence types outside of the reference database, though the proportions of these were small and decreased with increasing reference database size.

A best-genetic-match reference database approach (nearest neighbor) offers potential improvement over the ST reference database approach (lineage), in that it might improve the predictive performance for those classes that have a weaker association with a specific lineage (e.g., ceftriaxone, gentamicin, trimethoprim-sulfamethoxazole). The genetic distance approach seemed to operate best under two circumstances: (i) when only the top matches were considered and (ii) when a combined derivation data set was used. This top-match nearest-neighbor approach is potentially implemented using a predefined threshold of the Mash distance (or other genetic distance measure) but suffers from having a significant number of samples for which predictions may not be offered. Interestingly, the top-match approach can improve AUCs compared to those obtained by other approaches for the antibiotics that are less strongly associated with phenotype (ceftriaxone, gentamicin, trimethoprim-sulfamethoxazole).

One important consideration with a genetic distance-based model is the consideration of how large the reference database should be. Using repeated sampling methods, we found that optimal performance was achieved with comparatively small reference database sizes, consisting of 100 to 200 samples. This was consistent across the different classes of antibiotics tested, with plateaus in performance after ∼200 samples (Fig. S1), and consistent with previous work with other pathogens (10). However, the necessary size will likely depend on the diversity of the population being evaluated, with more diverse populations requiring larger databases. This is reflected in the performance seen with the combined derivation data sets.

There are limitations to our study. First, we were only able to consider the construction of reference databases confined to the geographic region of the province of Ontario, Canada. However, this region is geographically large and contains a population of over 14 million people. As such, our results still support construction of regional databases, at least at this jurisdictional level, which is generalizable to many areas globally. Second, we did not evaluate the utility of this approach for other bacterial species; however, E. coli is the most common Gram-negative pathogen in the hospital and community. Third, we did not examine in detail the reasons for failure to accurately predict a susceptibility phenotype. The potential reasons for the imperfect performance are numerous and include the comprehensiveness of the reference database, the acquisition of mobile genetic elements or new resistance mutations, human/labeling error, and imperfection in phenotypic testing methodologies. We did not seek to explore all of these reasons, but instead, we sought to quantify the overall additional benefit that these approaches could add. However, future work specifically exploring and characterizing the modes of failure is warranted. Other future work will aim to prospectively evaluate these techniques in a clinical setting using rapid sequencing approaches across geographic scales and additional pathogens. Our recent work suggests that we can predict susceptibility within minutes, and when this is combined with the use of rapid DNA extraction kits (<30 min) and rapid library preparation kits (<15 min), then it is currently feasible to go from clinical sample collection to a result in under 60 min (10). This time frame will likely shrink as DNA extraction and library preparation steps are further improved and simplified. In summary, our results suggest that rapidly obtainable genomic information from clinical isolates can support intelligent choices that improve empirical antibiotic therapy both by rescuing narrow-spectrum agents for therapeutic use and by better selecting broader-spectrum agents.

## MATERIALS AND METHODS

### Study design.

We performed a retrospective study to evaluate whether genetic relatedness can predict antibiotic susceptibility in E. coli isolates from 968 episodes of suspected and confirmed human infection. Three separate data sets were combined for this analysis and included data for 411 E. coli isolates from bloodstream infections at Sunnybrook Health Sciences Centre (SHSC), a single tertiary-care medical center in Toronto, Canada, collected over the years from 2010 to 2015 (data set 1), 177 E. coli isolates from suspected urinary tract infections from SHSC for the year 2018 (data set 2), and 380 multidrug-resistant (MDR) E. coli isolates from urinary sources from the Canadian province of Ontario collected in 2010 and 2015, where MDR was defined as resistance to at least three different classes of routinely tested antibiotics (data set 3).

### Resistance phenotype.

Antibiotic susceptibility phenotypes for ciprofloxacin (fluoroquinolones), trimethoprim-sulfamethoxazole (sulfonamides), ceftriaxone (3rd-generation cephalosporins), gentamicin (aminoglycosides), and ertapenem (carbapenems) were determined for each isolate using Vitek 2 AST cards. Clinical Laboratory Standards Institute (CLSI) 2015 breakpoints were employed for determining susceptible and nonsusceptible phenotypes for all data sets (see Table S1 in the supplemental material). For data set 2, only formal extended-spectrum-β-lactamase (ESBL) testing was available (ceftriaxone MICs were not reported), and as such, we classified all non-ESBL-producing E. coli isolates as susceptible to ceftriaxone. Given that we calculated susceptibility using MICs and static breakpoints for all data sets, there were no temporal changes in the interpretation of susceptibility. We considered all nonsusceptible isolates to be resistant throughout this study.

### Whole-genome sequencing.

Genomes for each data set were sequenced separately using a NextSeq high-output platform with Nextera library preparation with mean coverages of 134, 90, and 81 times for data sets 1, 2, and 3, respectively. Further sequencing details can be found in the methods in the supplemental material.

### Overall prediction approach.

As overfitting could be a major limitation of our approach and to simulate potential clinical implementation strategies, we externally validated previously collected derivation data sets (data sets 1 and 3) for predicting the susceptibility of isolates from the most recent data set (data set 2). Where applicable, we also evaluated the sets internally with bootstrapping to adjust for optimism (overfitting). We followed the general principles of the TRIPOD statement for reporting of multivariable prediction models (16).

Four prediction model approaches were employed and are described in detail in the supplemental material. Briefly, the first model was an ST parametric model approach which used ST (as a marker of the lineage) as a categorical predictor within a logistic regression model to predict the probability of susceptibility. The second model was a cluster parametric model approach that used labeled genetic clusters (as a marker of the lineage) as categorical predictors in a logistic regression model to predict the probability of susceptibility. The third model was an ST reference database approach that used the average prevalence of susceptibility to an antibiotic for a given ST (as a marker of the lineage) as the predicted probability of susceptibility. The fourth model was a best-genetic-match reference database approach that used the susceptibility phenotype of the best genetic match (nearest neighbor) in a reference database as the predicted susceptibility. The analyses described above were performed separately for each antibiotic.

To further simulate the antibiotic decision-making process, we explored two different scenarios, in which an antibiotic was selected based upon sequential model outputs either favoring narrow-spectrum agents or favoring a high likelihood of the adequacy of coverage. We called these sequential decision-making models. Further details on these methods are described in the supplemental material. Institutional research ethics board approval from SHSC was obtained for this study.

### Data availability.

Sequencing data have been made available through public databases and links to these data sets can be found in the supplemental material.

## Supplementary Material

Supplemental file 1
