# Supplementary material for: Using Genetic Distance from Archived Samples for the Prediction of Antibiotic Resistance in Escherichia coli
Source: Antimicrob Agents Chemother. 2020 Apr 21;64(5):e02417-19. doi: 10.1128/AAC.02417-19 (PMC7179619; doi:10.1128/AAC.02417-19)
Supplement: Supplemental file 1 [file AAC.02417-19-s0001.docx]

# **Supplementary Materials**

## **Supplementary Methods**

*Whole Genome Sequencing*

Sequence quality was assessed for each sample using FastQC (Version 0.11.5) and summarized with MultiQC (Version 1.7)[(1)](https://paperpile.com/c/Vf3VMQ/IJ8C9). Reads were trimmed with Trimmomatic (Version 0.36) to remove low-quality bases, using a sliding window of 4 and minimum read quality (phred) score of 15 [(2)](https://paperpile.com/c/Vf3VMQ/duuLK). Contigs were assembled using SPAdes (Version 3.8.0)[(3)](https://paperpile.com/c/Vf3VMQ/Rkofv). Kraken (Version 1.1 with minikraken 8Gb database) was used to confirm species identity from the contigs. Multi-locus sequence type (MLST) was identified *in silico* through the PubMLST database using the software package MLST [(4,5)](https://paperpile.com/c/Vf3VMQ/bPOpV+mR3Aa). For the minority of isolates without a sequence type (ST) identified from contigs, stringMLST[(6)](https://paperpile.com/c/Vf3VMQ/jPwQa) was performed from raw reads. Genes were annotated with Prokka (Version 1.13) [(7)](https://paperpile.com/c/Vf3VMQ/mk2wN). A common core genome for each individual dataset was identified using Roary (Version 3.12.0)[(8)](https://paperpile.com/c/Vf3VMQ/Ms04m) and clusters (groups of genetically related isolates) were identified and categorized using RhierBAPS (Version 1.1.0)(9). Genetic distances between isolates were calculated from contigs using Mash distances. Mash distance is a rapid measure of genetic distance that is based off of the minhash algorithm[(10)](https://paperpile.com/c/Vf3VMQ/zlg13). Genetic trees based on these Mash distances were generated using Mashtree (Version 0.57) [(10,11)](https://paperpile.com/c/Vf3VMQ/zlg13+6BcoA). Prediction models were developed using R (Version 3.5.2). Sequencing data is accessible through (1) the National Center for Biotechnology Information under the BioProject PRJNA521038, and through Zenodo.org under the DOIs: 10.5281/zenodo.3706855 and 10.5281/zenodo.3701595.

*ST Parametric Model Approach (Lineage-based)*

For the entire dataset, we developed logistic regression models to predict antibiotic susceptibility, given as susceptible (1) or non-susceptible (0). The predictor variables consisted of binary indicator variables of the presence of specific STs. Nine of the most common STs were considered as binary variables, with the final 10^th^ variable representing the remaining STs with the lowest frequencies. For derivation and validation datasets, the area under the curve (AUC) for the receiver operating characteristic (ROC) curves was computed with 95% confidence intervals. The area under the curve represents a measure of the model to discriminate between different outcomes, e.g. do isolates with high predicted likelihood of resistance have a higher true prevalence of resistance than those isolates with a low predicted likelihood of resistance. The closer the AUC is to 1, the better the ability to discriminate between outcomes. Random guessing would generate an AUC of 0.5. AUC’s are often considered insensitive measures of discrimination, and even small changes in magnitude of AUC can indicate potential clinical significance. For internal validation of individual derivation datasets, bootstrapping (1000 samples with replacement) was performed in order to estimate optimism-corrected (adjusted) AUCs. 0.6% of sampled isolates had STs that could not be identified (either because of assembly quality or because they were not present in the PubMLST database) and were classified with the group of ‘remaining’ STs for categorization.

*Cluster Parametric Model Approach (Lineage-based)*

Similar to the ST parametric approach, we also developed logistic regression models to predict antibiotic susceptibility but using clusters as the predictors. The predictors (clusters of genetically related isolates) were identified as described above, and consisted of binary indicator variables for the presence of a specific cluster ID. We limited the maximum number of identified clusters to 10, for comparability with the *ST Parametric Model Approach*, and to ensure model fit/convergence. For derivation and validation datasets, the area under the curve (AUC) for the receiver operating characteristic (ROC) curves was computed with 95% confidence intervals. Since the clustering could only be performed within an individual dataset, and was not comparable between datasets (unlike the ST approach), we did not perform external validation, but rather performed internal validation for each dataset. Bootstrapping (1000 samples with replacement) was performed in order to estimate optimism-corrected (adjusted) AUCs for each dataset.

*ST Reference Database Approach (Lineage-based)*

In addition to the above *ST and Cluster Parametric Models,* we also employed a reference database approach for each dataset. This consisted of creating an antibiotic susceptibility reference database, whereby we determined the prevalence of susceptibility to each antibiotic for each sequence type for a given dataset. Creating, in essence, an ST-specific antibiogram that could be used to predict susceptibility. Because of the inherent issue of overfitting with this approach, we only validated a given dataset (Dataset 1, 3, or 1 and 3) with a separate dataset (Dataset 2). Missing STs were combined together into a single category in the reference index. This reference database approach offers the advantage of providing antibiotic susceptibility phenotype predictions for rarer STs, which collectively make up a reasonable proportion of the total observed diversity in the clinical setting.

*Best Genetic Match Reference Database Approach (Nearest neighbor)*

We sought to test whether antibiotic susceptibility could be best predicted using the closest genetically matched isolate within the reference database, as opposed to a higher level lineage. To do this, we created a reference database of the best genetic match for each isolate and assigned predicted antibiotic susceptibility phenotype based on the phenotype of the best genetic match (shortest Mash distance). To evaluate whether this approach performed better when the reference database contained a closely related isolate than when it did not, we reported overall results as well as results considering only the top 25%’ile and bottom 75%’ile of similarity between the query strain and the closest strain in the reference database.

*Model Performance*

Test characteristics including sensitivity, specificity, negative predictive value (NPV), and positive predictive value (PPV) were calculated, and in the case of continuous predictors, the optimal ROC threshold (by Youden’s J statistic) was used (pROC package, R)[(12)](https://paperpile.com/c/Vf3VMQ/t1xa1). Since susceptibility to ertapenem in the validation dataset (Dataset 2) was 100%, we could not calculate model performance through external validation, however we have instead provided the internally validated performance of the model (for *ST and Cluster Parametric Models* approaches only) in the supplemental material (Supplementary Table 2). Moreover, we can still use the ertapenem models for predicting antibiotic selection in a *Sequential Decision-making Model* (see below).

*Summary of Test Characteristics Across Models*

We generated summary figures for the post-test probabilities of susceptibility for: (1) positive model predictions indicating a likely susceptible isolate (PPV [positive predictive value]); and (2) for negative model predictions indicating a likely resistant isolate (1-NPV [Negative predictive value]), for all antibiotics and models. For antibiotics with a low pre-test probability of susceptibility (e.g., ciprofloxacin and trimethoprim-sulfamethoxazole), the goal is to obtain post-test probabilities (for model results indicating susceptible) shifted to the right (and specifically to the right of syndrome-specific thresholds considered adequate by the prescriber). This would reflect an improved ability to predict susceptibility and the opportunity to use these agents empirically. For antibiotics with a generally high pre-test probability of susceptibility (e.g. ceftriaxone, gentamicin, ertapenem), the goal is to obtain post-test probabilities (for model results indicating resistant) shifted to the left (and specifically to the left of syndrome-specific thresholds), which would reflect an improvement in our ability to predict resistance and the opportunity to prevent inadequate prescribing. We assumed that 80% and 90% prevalence of susceptibility were important thresholds for low and high morbidity syndromes respectively (13-14)

*Sequential Antibiotic Decision-Making Based Upon a Prespecified Antibiotic Cascade*

To simulate the antibiotic decision-making process, we explored two different scenarios where an antibiotic was selected based upon model outputs, either favouring narrow spectrum agents (Narrow spectrum cascade: ciprofloxacin → trimethoprim-sulfamethoxazole → ceftriaxone → gentamicin → ertapenem) or favouring adequacy (Adequacy cascade: ertapenem → gentamicin → ceftriaxone → trimethoprim-sulfamethoxazole → ciprofloxacin). Here the narrow spectrum cascade is ordered from narrowest to broadest spectrum, and the adequacy cascade is ordered from highest susceptibility against *E. coli* to lowest, in this dataset. The particular order of these cascades could also be tailored to desired outcome, e.g. prioritizing the use of agents that are less expensive or less likely to cause *Clostridioides difficile* infection. To simulate the sequential selection process, the first model for a given antibiotic in a cascade was run, and if susceptibility was predicted, then this antibiotic was chosen. Otherwise the next antibiotic model was queried. For the models generating a probabilistic prediction, a threshold of 80% susceptibility was used to prompt recommendation of an antibiotic[[13]](https://paperpile.com/c/Vf3VMQ/rq44), though this could be adjusted[[14]](https://paperpile.com/c/Vf3VMQ/tjvxg). We employed the *ST Reference Database Approach*, the *Best Genetic Match Reference Database*, and combinations of the two models in this sequential decision-making simulation. To test whether the narrow spectrum cascade improved prescribing, a spectrum score was generated similar to previously described [[14]](https://paperpile.com/c/Vf3VMQ/tjvxg). We created a simple ordinal score (normalized by the maximum possible value) in the following fashion: we assigned the lowest spectrum antibiotic (ciprofloxacin) to a weight of 0, then the next lowest spectrum antibiotic trimethoprim-sulfamethoxazole to a weight of 1, then the next lowest spectrum antibiotic (ceftriaxone) to a weight of 2, and so on according to the narrowest to broadest spectrum cascade. With this score, the highest possible value (corresponding to 100% of patients receiving ertapenem therapy) is 1 and the lowest possible value is 0 (corresponding to 100% of patients receiving ciprofloxacin). This score makes the arbitrary assumption of linear increases in the magnitude of spectrum as one moves along the spectrum cascade, but is illustrative for summarizing spectrum across treatment recommendation approaches.

## **Supplementary Tables**

**Supplementary Table 1. Susceptibility breakpoints (μg/mL) by antibiotic and dataset.
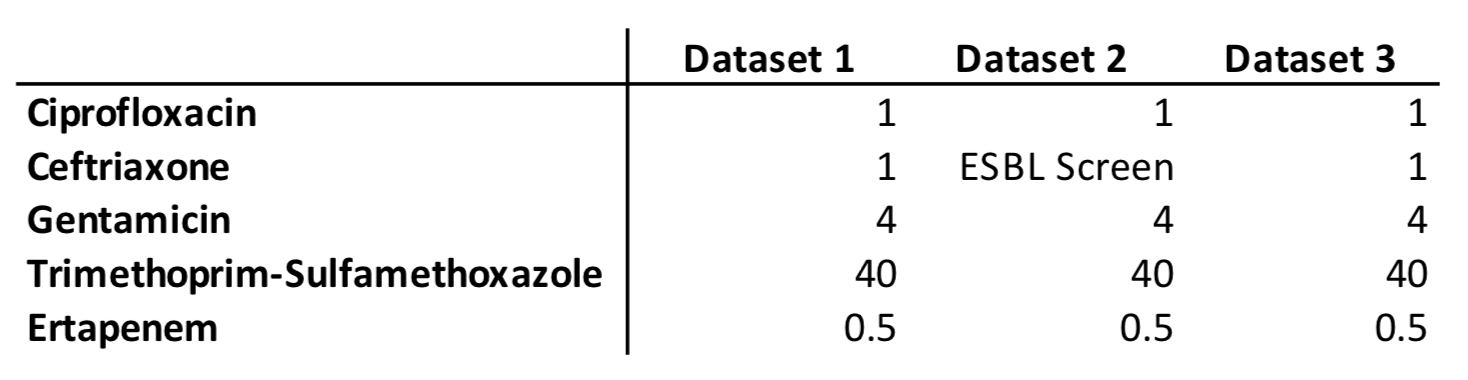
**

**Supplementary Table 2. Receiver operating characteristic area under the curve (AUC) and test characteristics of predicting susceptibility to ertapenem, using internal validation and bootstrapping to correct for optimism. (A) *ST Parametric Model Approach* and (B) *Cluster Parametric Model Approach*.**

(A)

**
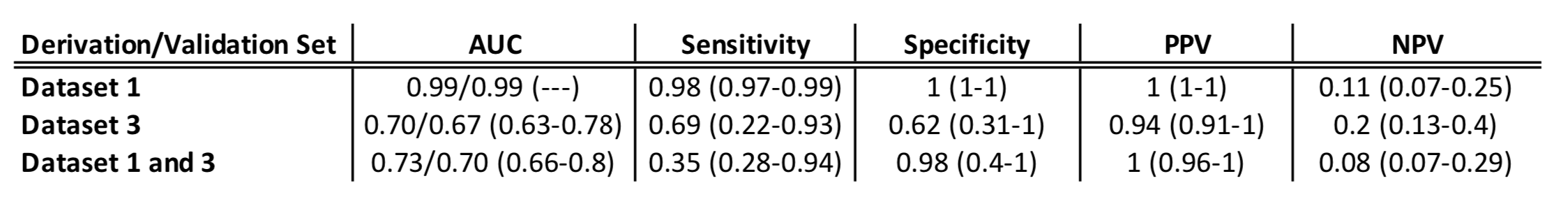
**

(B)

**
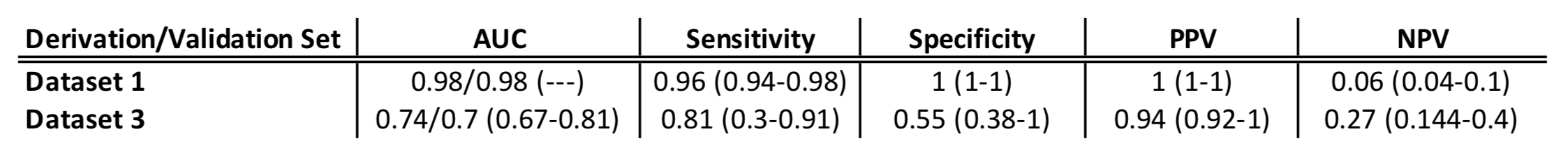
**

*Bootstrapped optimism adjusted AUCs are shown for internally validated sets as AUC/Adjusted AUC. Confidence intervals could not be calculated for Set 1 AUC.

**Supplementary Table 3. Receiver operating characteristic area under the curve (AUC), and test characteristics of predicting antibiotic susceptibility of Dataset 2 using an *ST Parametric Model Approach*. Ciprofloxacin (A), ceftriaxone (B), gentamicin (C), trimethoprim-sulfamethoxazole(D).**

(A)


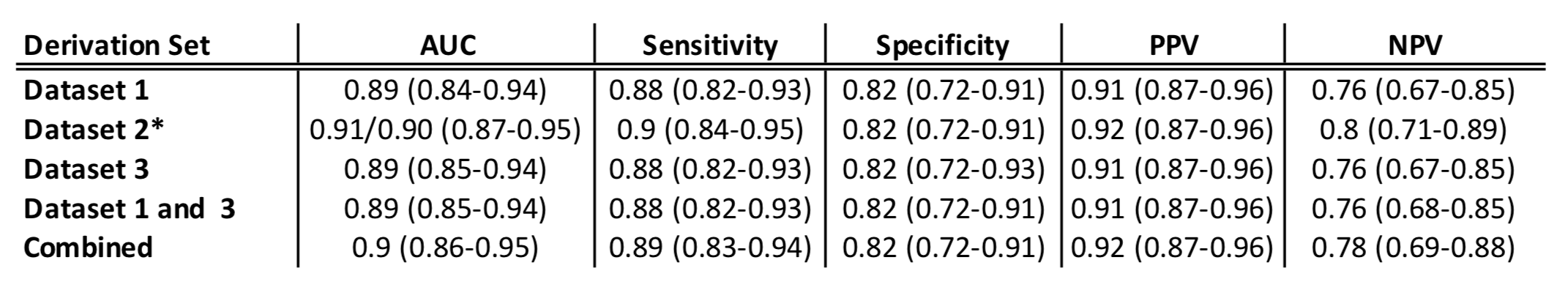


(B)


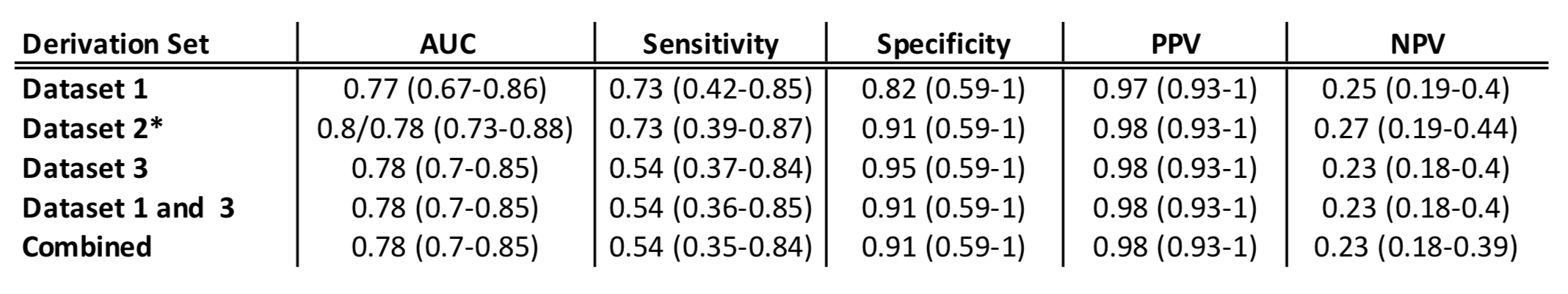


(C)


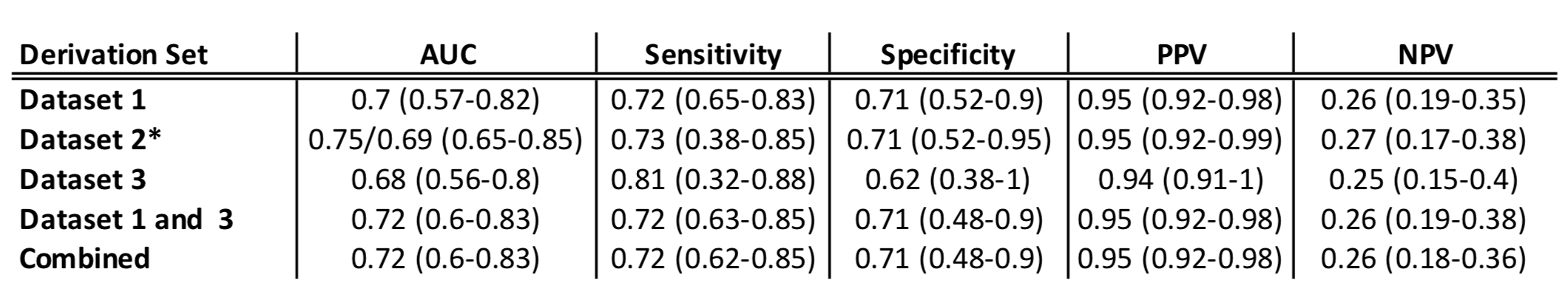


(D)


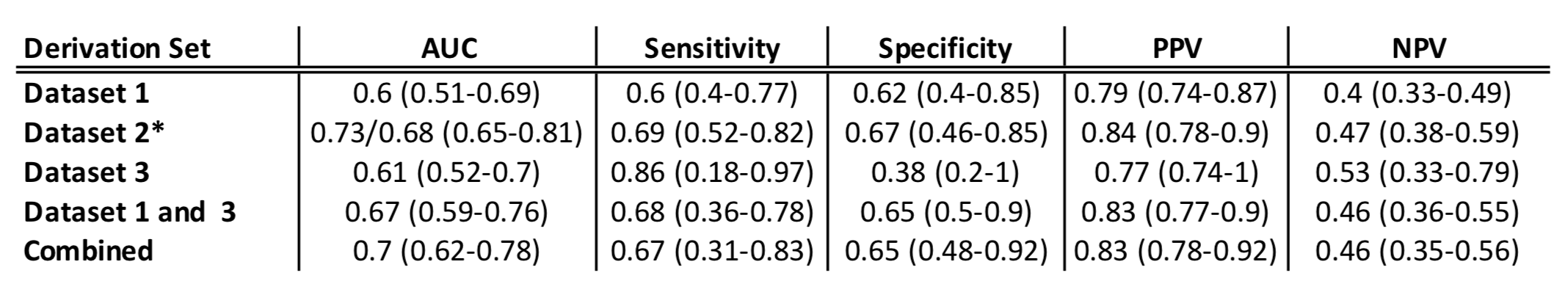


*Bootstrapped optimism adjusted AUCs are shown for internally validated sets as AUC/Adjusted AUC.

**Supplementary Table 4. Internally validated receiver operating characteristic area under the curve (AUC), and test characteristics of predicting antibiotic susceptibility (internally) using a *Cluster Parametric Model Approach*. Ciprofloxacin (A), ceftriaxone (B), gentamicin (C), trimethoprim-sulfamethoxazole (D).**

(A)


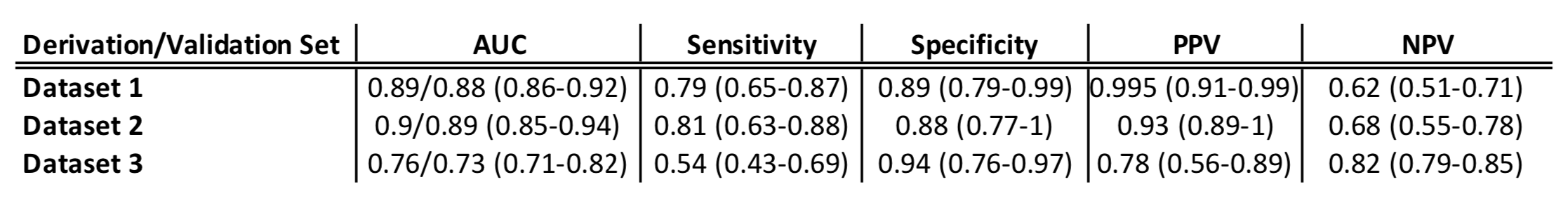


(B)


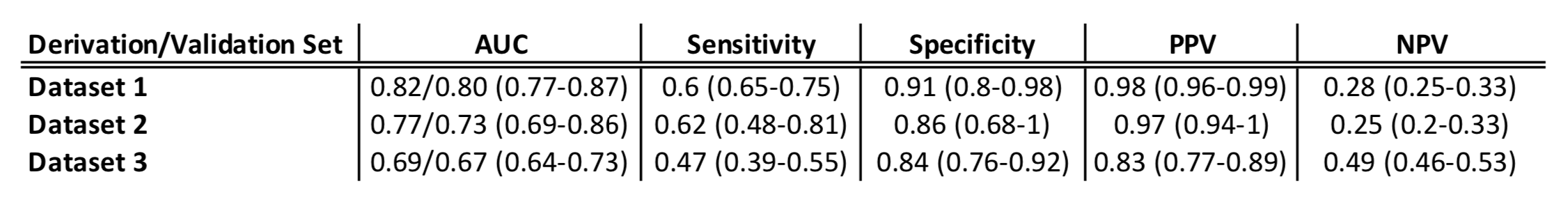


(C)


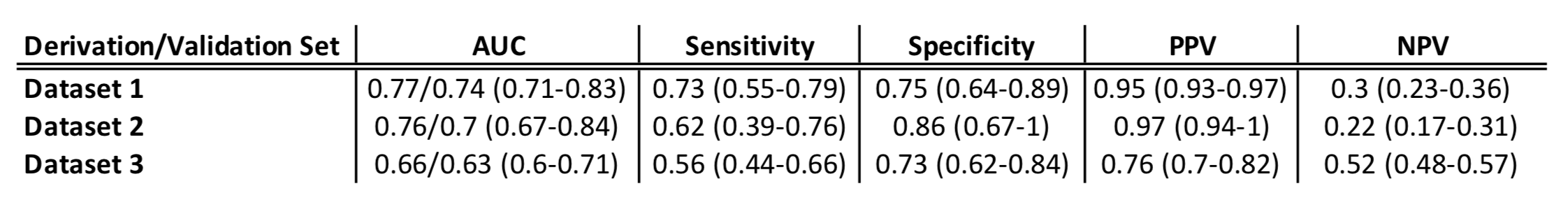


(D)


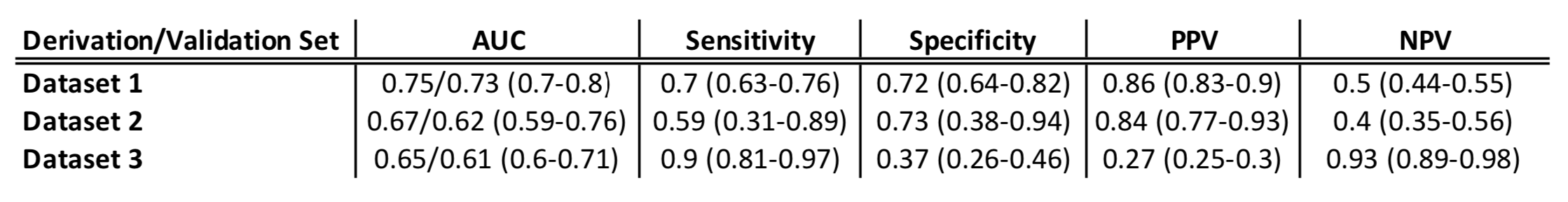


*Bootstrapped optimism adjusted AUCs are shown as AUC/Adjusted AUC.

**Supplementary Table 5. Receiver operating characteristic area under the curve (AUC), and test characteristics of predicting antibiotic susceptibility of isolates in Set 2 using an *ST Reference Database Approach*. Ciprofloxacin (A), ceftriaxone (B), gentamicin (C), trimethoprim-sulfamethoxazole (D). N missing indicates the number of samples in the validation set that could not be matched to an ST in the reference database.**

(A)
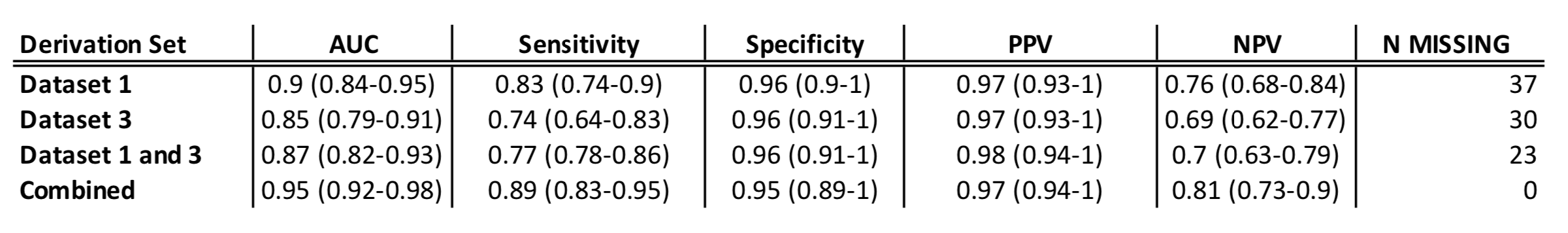


(B)


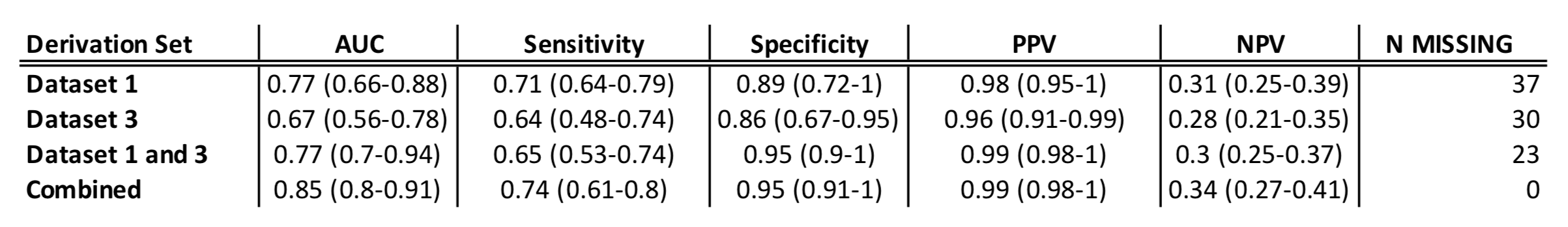


(C)


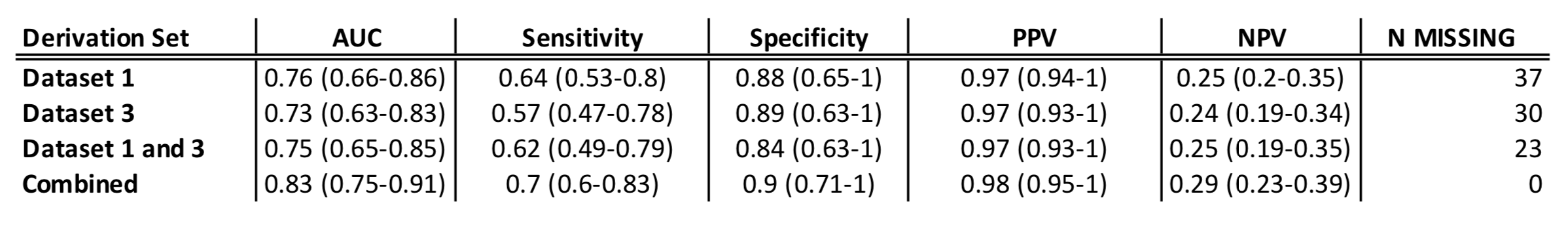


(D)


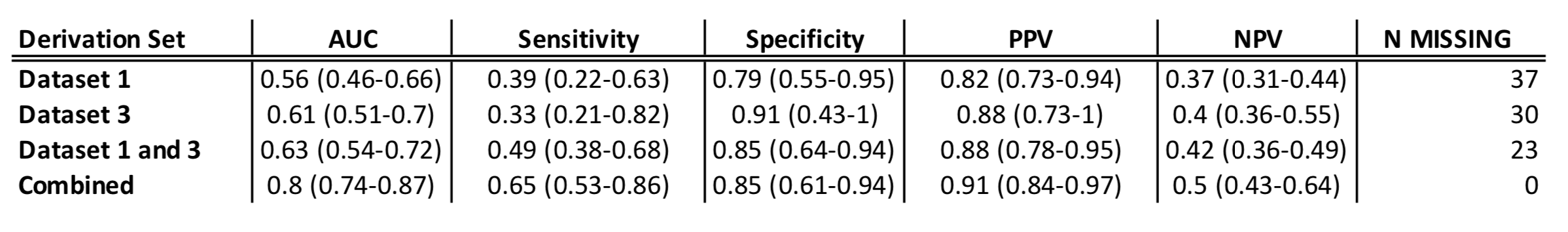


**Supplementary Table 6. Receiver operating characteristic area under the curve (AUC), and test characteristics of predicting antibiotic susceptibility of Set 2 using a *Best Genetic Match Reference Database* (shortest Mash distance). Models were also stratified by the quartile with the shortest mash distances (Top). Ciprofloxacin (A), ceftriaxone (B), gentamicin (C), trimethoprim-sulfamethoxazole (D). ‘Sens prevalence’ corresponds to the prevalence of susceptibility to the given antibiotic for a specific subset.**

(A)

*All Isolates*

*
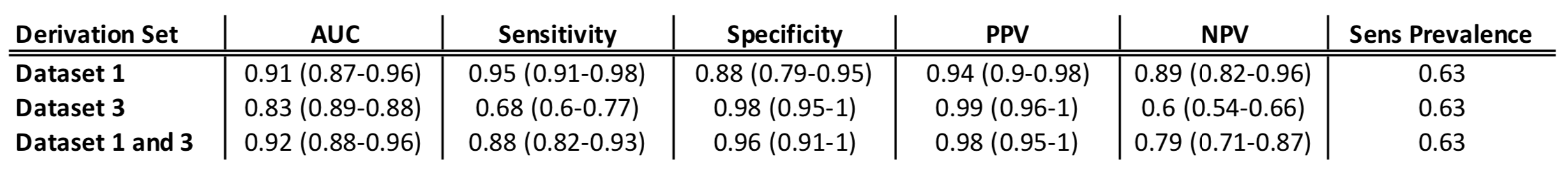
*

*25% Top Match (Shortest Mash distances)*


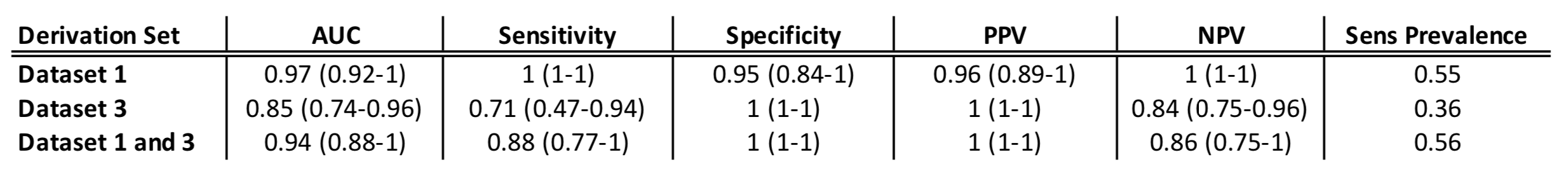


*75% Bottom Match (Longest Mash distances)*


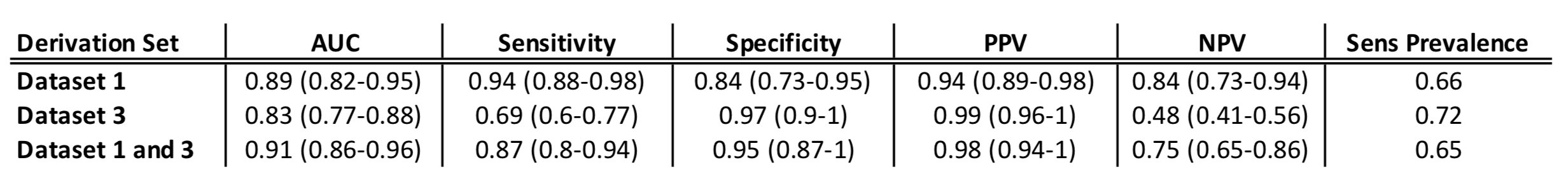


(B)

*All Isolates*


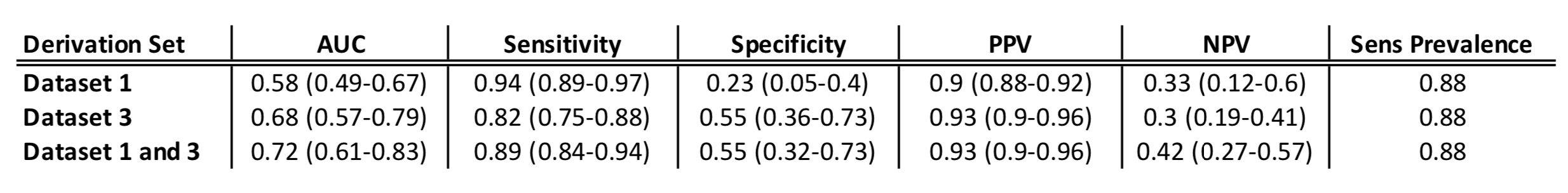


*25% Top Match (Shortest Mash distances)*


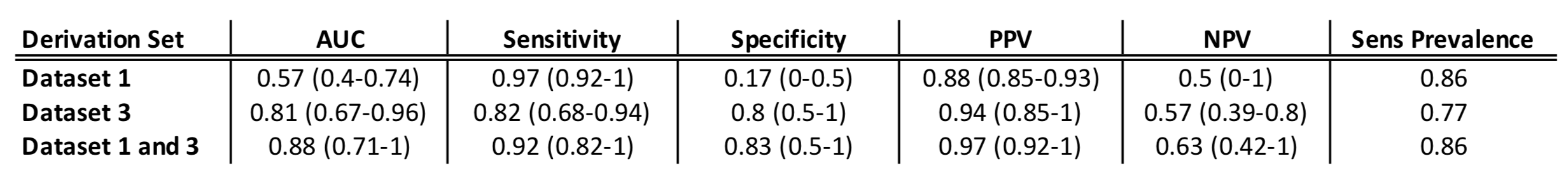


*75% Bottom Match (Longest Mash distances)*


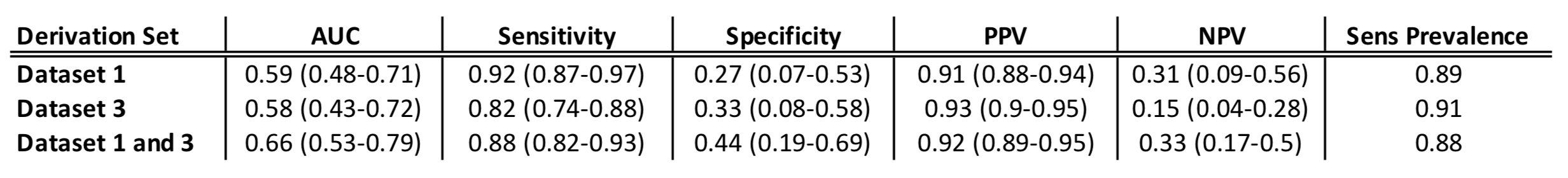


(C)

*All Isolates*


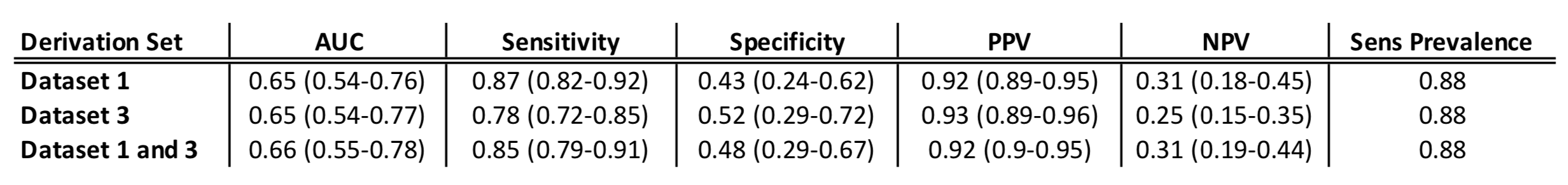


*25% Top Match (Shortest Mash distances)*


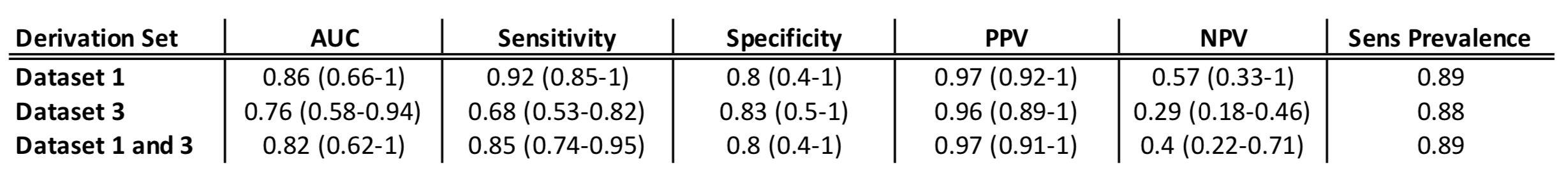


*75% Bottom Match (Longest Mash distances)*

*
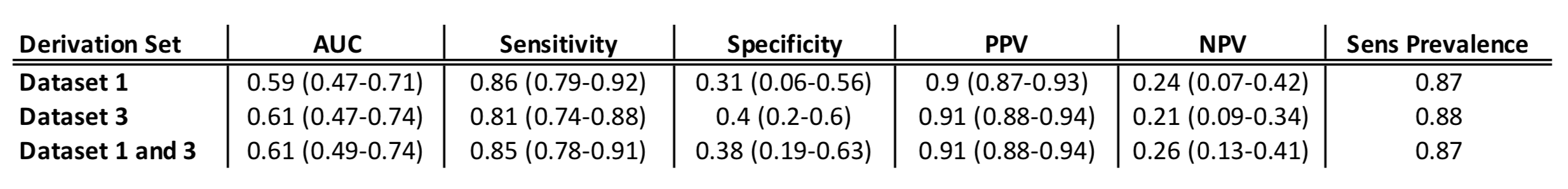
*

(D)

*All Isolates*


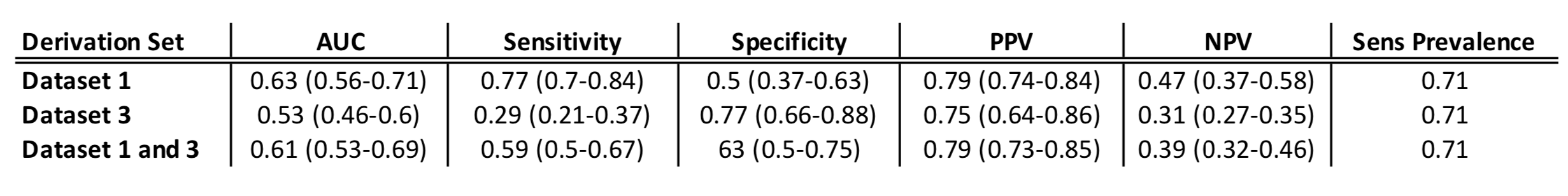


*25% Top Match (Shortest Mash distance)*


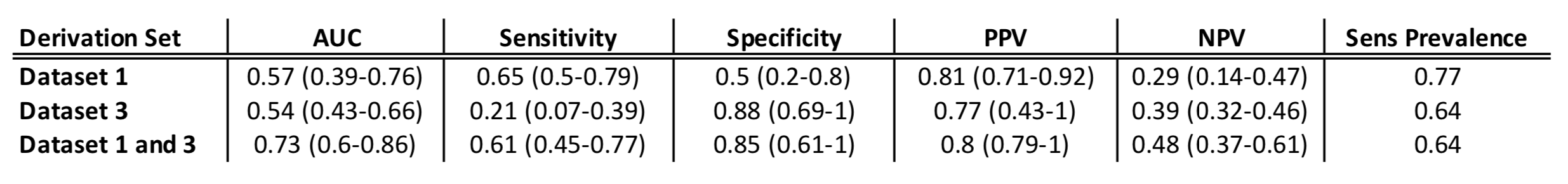


*75% Bottom Match (Longest Mash distance)*


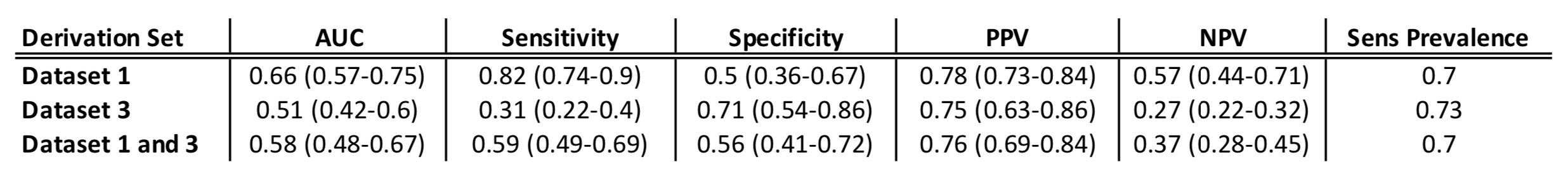


**Supplementary Table 7. Proportion of antimicrobial use by antibiotic, adequacy, oral administration option, and spectrum score, for Dataset 2.**

**
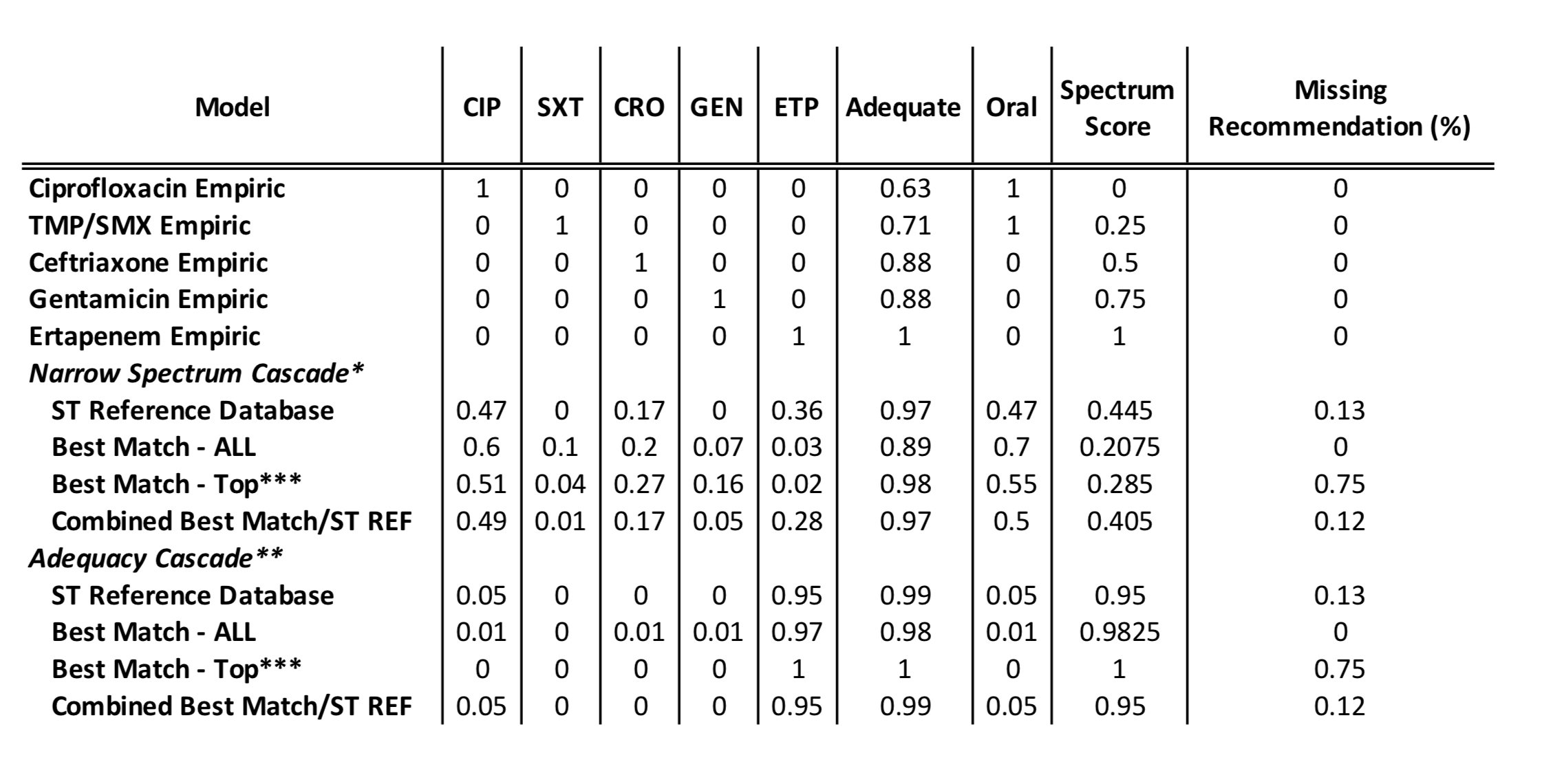
**

*Narrow spectrum cascade: Cipro->Septra->Ceftriaxone->Gentamicin
**Adequacy cascade: Ertapenem->Gentamicin->Ceftriaxone->Septra->Ciprofloxacin
***TOP 25%'tile of Best Match, and 80% treatment threshold used for continuous predictors.

## **Supplementary Figures**

**Supplementary Figure 1. Average receiver operating characteristic area under the curve (AUC), from 250 bootstraps, as a function of randomly selected without replacement derivation databases of varying sizes tested in Dataset 2. Ciprofloxacin (A), ceftriaxone (B), gentamicin (C), trimethoprim-sulfamethoxazole (D).**

(A)


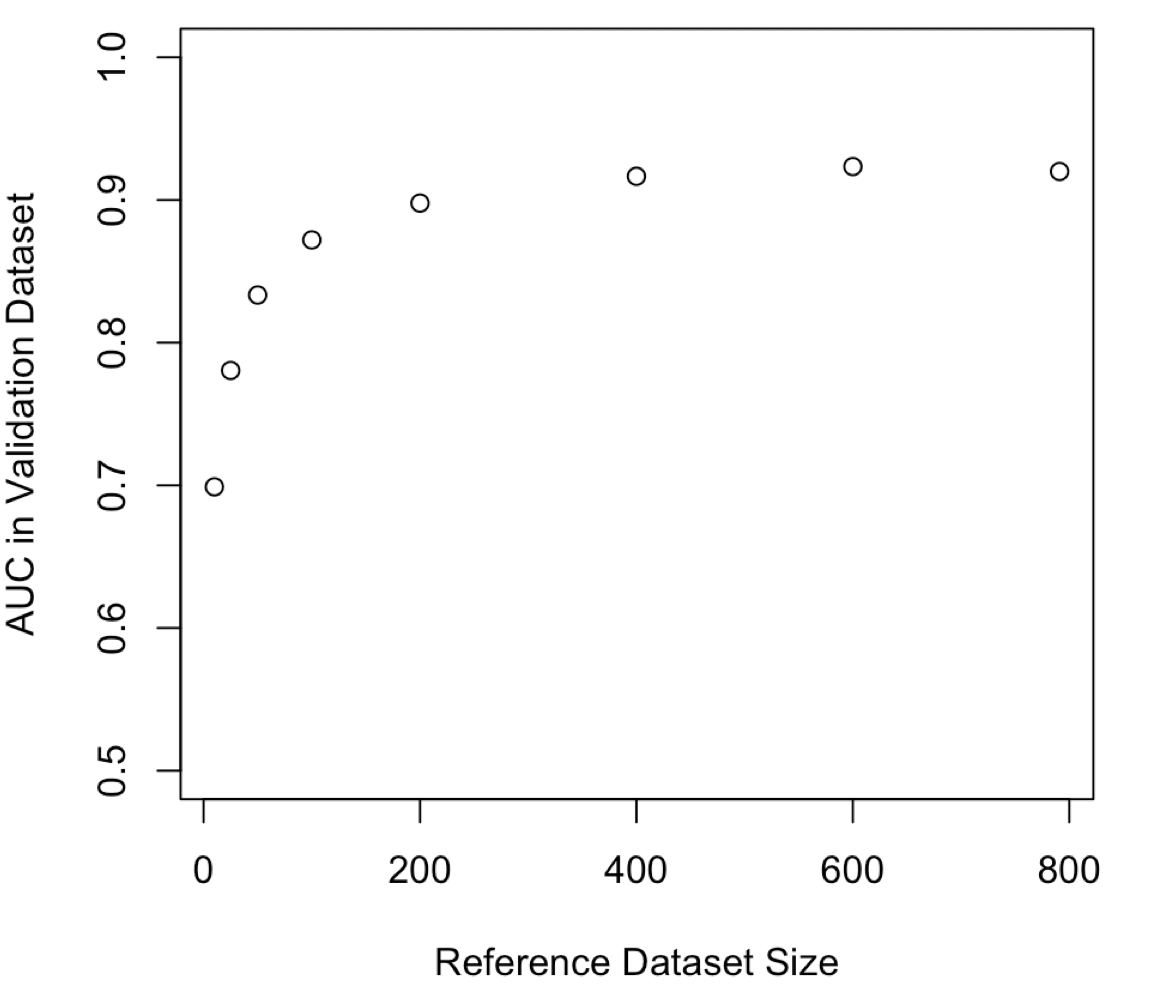


(B)


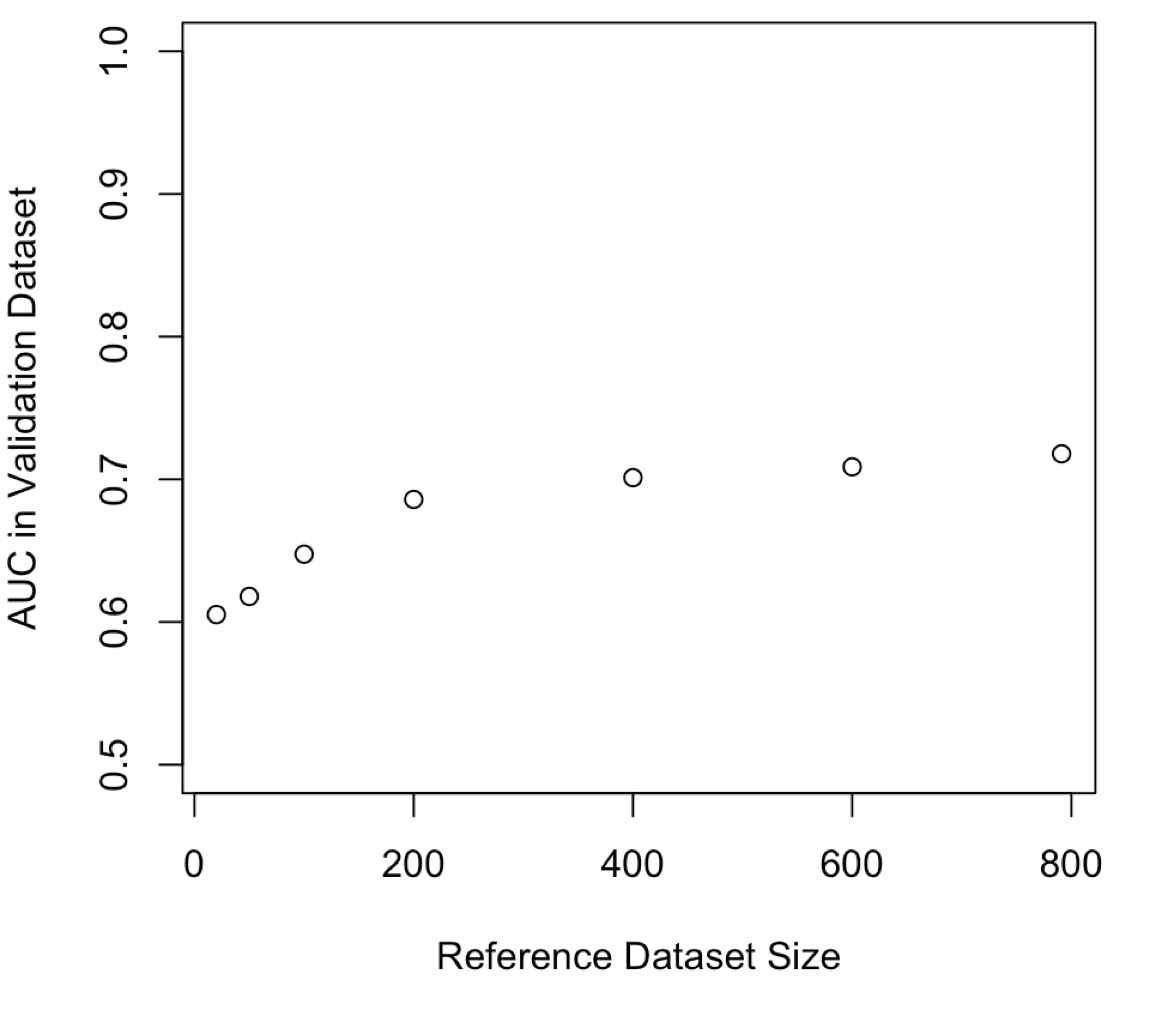


(C)


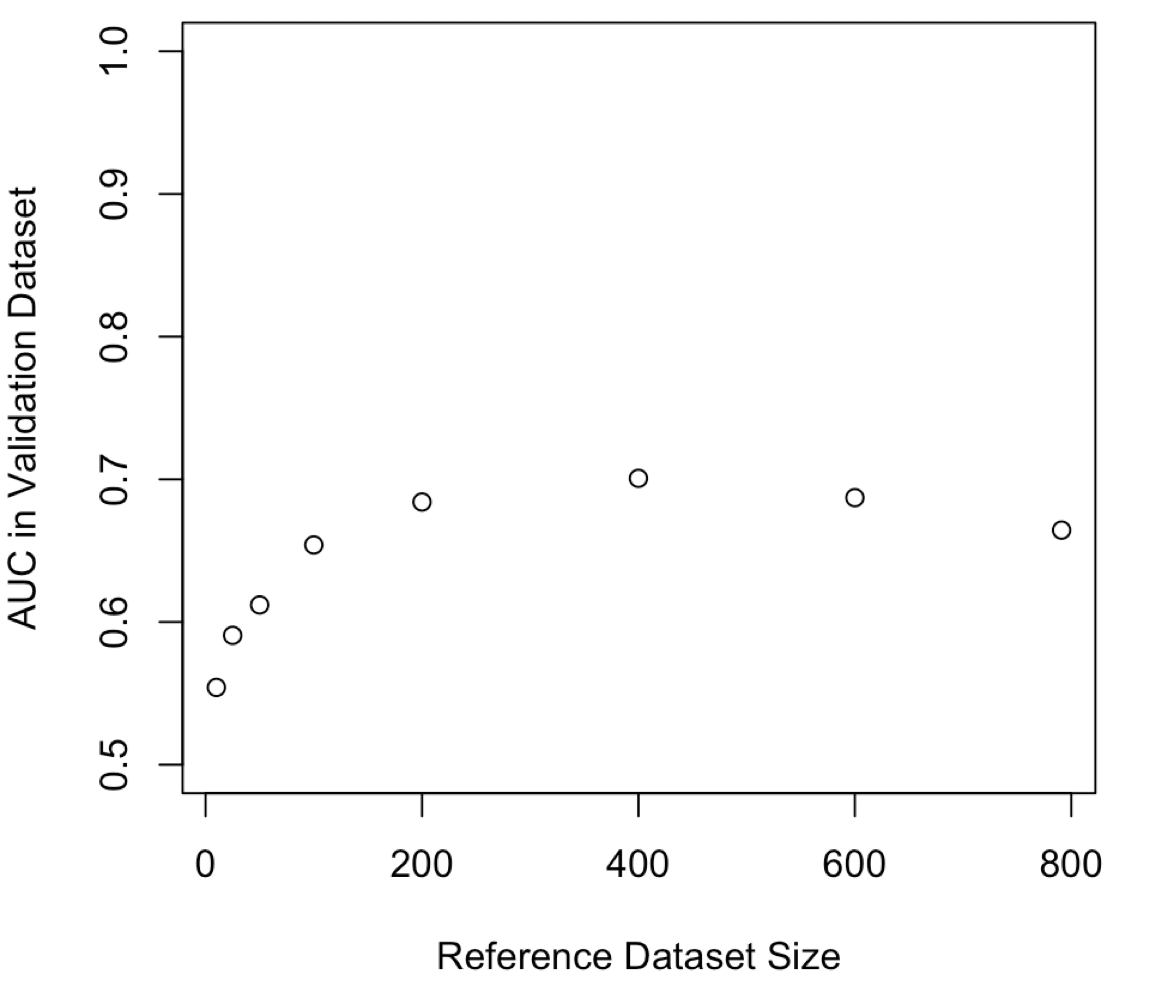


(D)


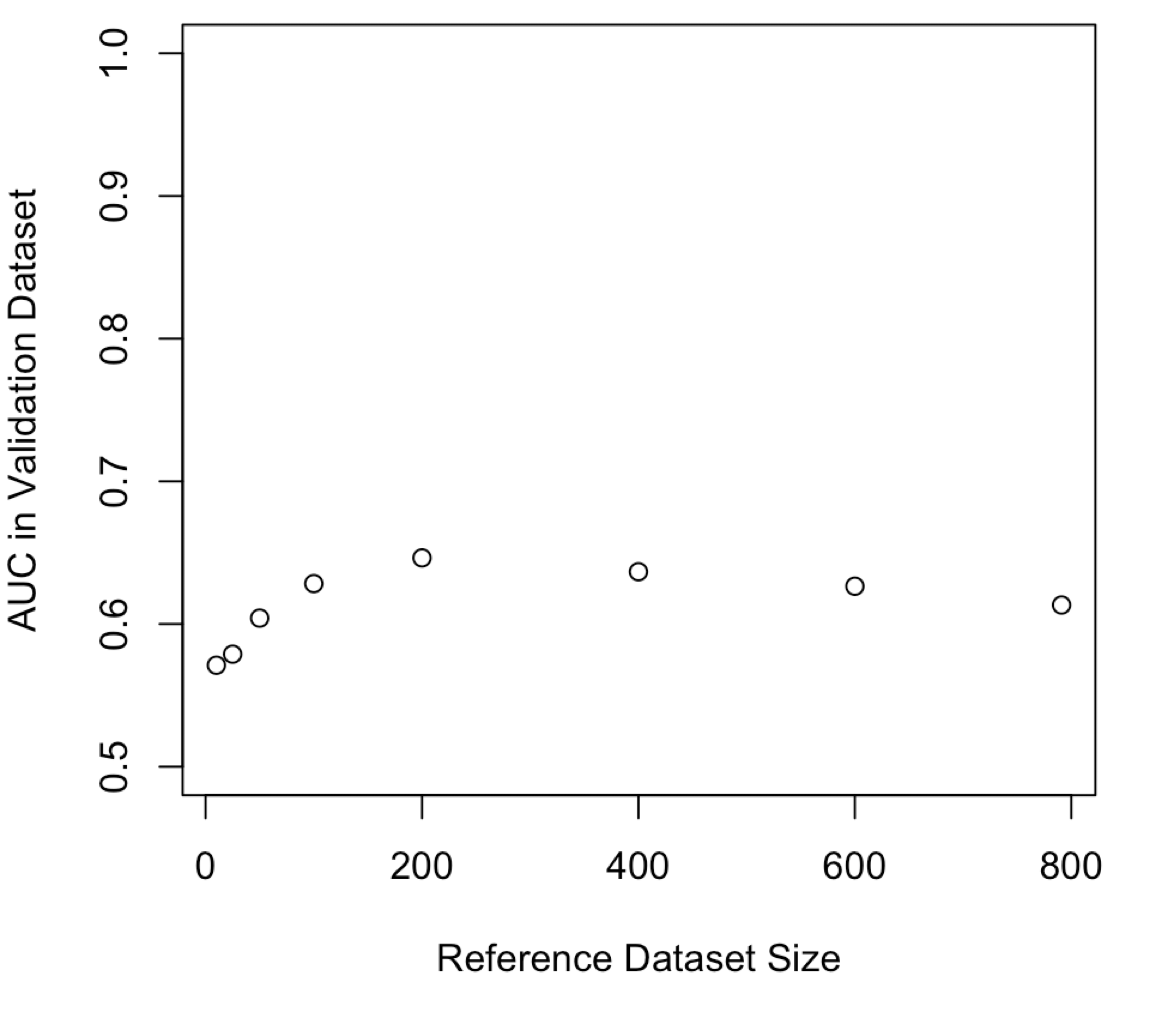


**Supplementary References**

1. Ewels P, Magnusson M, Lundin S, Käller M. MultiQC: summarize analysis results for multiple tools and samples in a single report. 2016. Bioinformatics 32:3047–3048. Available at: http://dx.doi.org/10.1093/bioinformatics/btw354.

2. Bolger AM, Lohse M, Usadel B. 2014. Trimmomatic: a flexible trimmer for Illumina sequence data. Bioinformatics 30:2114–2120.

3. Bankevich A, Nurk S, Antipov D, Gurevich AA, Dvorkin M, Kulikov AS, Lesin VM, Nikolenko SI, Pham S, Prjibelski AD, Pyshkin AV, Sirotkin AV, Vyahhi N, Tesler G, Alekseyev MA, Pevzner PA. 2012. SPAdes: a new genome assembly algorithm and its applications to single-cell sequencing. J Comput Biol 2012 19:455–477.

4. Jolley KA, Maiden MCJ. 2010. BIGSdb: Scalable analysis of bacterial genome variation at the population level. BMC Bioinformatics 11:595.

5. Seemann T. MLST. Available at: https://github.com/tseemann/mlst. Accessed 13 August 2019.

6. Gupta A, Jordan IK, Rishishwar L. 2017. stringMLST: a fast k-mer based tool for multilocus sequence typing. Bioinformatics 33:119–121.

7. Seemann T. 2014. Prokka: rapid prokaryotic genome annotation. Bioinformatics 30:2068–2069.

8. Page AJ, Cummins CA, Hunt M, Wong VK, Reuter S, Holden MT, Fookes M, Falush D, Keane JA, Parkhill J. 2015. Roary: rapid large-scale prokaryote pan genome analysis. Bioinformatics 31:3691–3693.

9. Tonkin-Hill G, Lees JA, Bentley SD, Frost SDW, Corander J. 2018. RhierBAPS: An R implementation of the population clustering algorithm hierBAPS. Wellcome Open Research. 3:93. Available at: http://dx.doi.org/10.12688/wellcomeopenres.14694.1.

10. Ondov BD, Treangen TJ, Melsted P,Mallonee A, Bergman N, Koren S, Phillippy A. 2016. Mash: fast genome and metagenome distance estimation using MinHash. Genome Biol 17:132.

11. Katz L. Mashtree. Available at: https://github.com/lskatz/mashtree. Accessed 13 August 2019.

12. Robin X. Package ‘pROC’. Available at: https://cran.r-project.org/web/packages/pROC/pROC.pdf. Accessed 14 August 2019.

13. Cressman AM, MacFadden DR, Verma AA, Razak F, Daneman N. 2018. Empiric Antibiotic Treatment Thresholds for Serious Bacterial Infections: A Scenario-Based Survey Study. Clin Infect Dis Available at: http://dx.doi.org/10.1093/cid/ciy1031.

14. MacFadden DR, Coburn B, Shah N, Robicsek A, Savage R, Elligsen M, Daneman N. 2019. Decision-support models for empiric antibiotic selection in Gram-negative bloodstream infections. Clin Microbiol Infect 25:108.e1–108.e7.
